# Acceptance of Digital Sports: A Study Showing the Rising Acceptance of Digital Health Activities Due to the SARS-CoV-19 Pandemic

**DOI:** 10.3390/ijerph19010596

**Published:** 2022-01-05

**Authors:** Jacqueline Ruth, Steffen Willwacher, Oliver Korn

**Affiliations:** 1 Department of Media and Information, Offenburg University of Applied Sciences, 77652 Offenburg, Germany; jruth@stud.hs-offenburg.de; 2 Department of Mechanical and Process Engineering, Offenburg University of Applied Sciences, 77652 Offenburg, Germany; steffen.willwacher@hs-offenburg.de; 3 Affective & Cognitive Institute, Offenburg University of Applied Sciences, 77652 Offenburg, Germany

**Keywords:** digital sports, pandemic, fitness app, augmented reality, gamification, human-computer interaction, mixed reality, wearable

## Abstract

In pandemic times, the possibilities for conventional sports activities are severely limited; many sports facilities are closed or can only be used with restrictions. To counteract this lack of health activities and social exchange, people are increasingly adopting new digital sports solutions—a behavior change that had already started with the trend towards fitness apps and activity trackers. Existing research suggests that digital solutions increase the motivation to move and stay active. This work further investigates the potentials of digital sports incorporating the dimensions gender and preference for team sports versus individual sports. The study focuses on potential users, who were mostly younger professionals and academics. The results show that the SARS-CoV-19 pandemic had a significant negative impact on sports activity, particularly on persons preferring team sports. To compensate, most participants use more digital sports than before, and there is a positive correlation between the time spent physically active during the pandemic and the increase in motivation through digital sports. Nevertheless, there is still considerable skepticism regarding the potential of digital sports solutions to increase the motivation to do sports, increase performance, or raise a sense of team spirit when done in groups.

## 1. Introduction

Sports and daily exercise are of enormous importance for maintaining body mobility and mental health in the long term. The contact restrictions required in pandemic times significantly reduce the possibilities for conventional indoor sports activities and even for outdoor team sports. Gyms are closed or limited to medical training. Working from home reduces movement even further: the daily commute to work and the corresponding walking routines on the company premises are eliminated. Overall, these restrictions result in drastic reductions in physical activity observed worldwide [1].

Digital health, defined as “the use of digital technologies for health” [2], is increasingly recognized as a major driver of quality in healthcare. A recent meta-analysis [3] exemplifies how such solutions can tremendously increase the quality of information and thus quality of treatment. At the same time, digital solutions aimed at sports and prevention are very successful; fitness trackers and smartwatches have already been established on the market for several years. Like their professional counterparts in the health domain, such devices monitor daily activities and exercises. Furthermore, they are designed to motivate persons to increase health activities and sports in the long term, such as by employing gamification elements. In pandemic times, solutions for digital health gain even more importance. 

To increase engagement, digital sports solutions for home use often employ augmented reality (AR), virtual reality (VR), or even mixed reality (MR) technologies, which previously have found little use in private households outside of the gaming domain. AR enriches the real world with virtual elements, which overlay the real world to generate live information or create immersion. In VR applications, on the other hand, users become entirely immersed in the virtual world. In professional sports, this technology offers the possibility of recreating stressful situations, such as large crowds and competitive situations. MR is a hybrid of AR and VR. The definition of MR can vary, but it typically describes a combination of AR and VR, allowing virtual objects to be anchored and interacted with in the real world [4]. This allows the use of tools such as special glasses and gloves. For example, at CES 2019 in Las Vegas, virtual training for field hockey players was presented. A significant advantage of such mixed reality training is that it minimizes the risk of injury, as interaction occurs exclusively with virtual opponents [5].

Recent work from Wilke et al. and others shows an increasing interest in “digital home training” in times of pandemic [1]. Based on the theory of planned behavior (TPB, see Section 2.3), we investigate how the pandemic influenced the acceptance of digital sports, especially in terms of behavioral control and social norms. To explore and quantify these effects and to see if there is a long-term change in behavior and norms, we conducted a study. Our underlying main hypotheses are:

**Hypothesis** **1** **(H1).** *Users who have stayed active during the pandemic also use more digital sports solutions.*

**Hypothesis** **2** **(H2).** *Persons using digital sports solutions aim to continue using these in the long run.*

The work is structured into five sections. First, we briefly investigate overall trends in digital sports and examine three popular best practices. We then introduce basic concepts regarding technology acceptance and discuss related studies, often focusing on the impact of the pandemic on physical activity, health, and mental well-being. In the study, we analyze and discuss how the 130 mostly young participants changed their views on digital sports because of the pandemic and discuss the results. We particularly look at how gender and the preference for individual sports or team sports affects the acceptance of digital sports. The work concludes with a summary of the essential findings and a discussion of limitations and future work.

## 2. Best Practices in Digital Sports

This section first portrays current digital sports solutions for private households, focusing on fitness apps and fitness trackers. We then discuss three best practices in digital sports that have gained prominence because of the coronavirus pandemic. 

### 2.1. Digital Technologies in the Fitness and Health Market

In the Worldwide Survey of Fitness Trends 2021 of the ACSM’s Health & Fitness Journal, a total of four digital technologies are in the top 20 ranking of all fitness trends surveyed. Online training went from twenty-sixth place in 2020 to first place in 2021. According to the survey, this can be attributed to the shift in the market caused by the pandemic. Wearable technology, after two years at the first position, is now ranked second. Virtual training is ranked sixth, and fitness apps twelfth [6].

Indeed, digital fitness apps, fitness trackers, and smartwatches have been established in the market for several years and are gaining popularity. Fitness trackers and smartwatches are called wearables, as they are worn on the body. These wearables are equipped with various sensors, such as accelerometers, gyroscopes, and barometers. These sensors determine health-related data, such as number of steps, energy consumption, or sleep rhythm. Furthermore, the heart rate can be measured and reported via photoplethysmography [7]. Training sessions can be recorded, and it is possible to track nutritional behaviors. In most cases, the generated data are then evaluated and visualized via the fitness app. 

**Figure 1:**
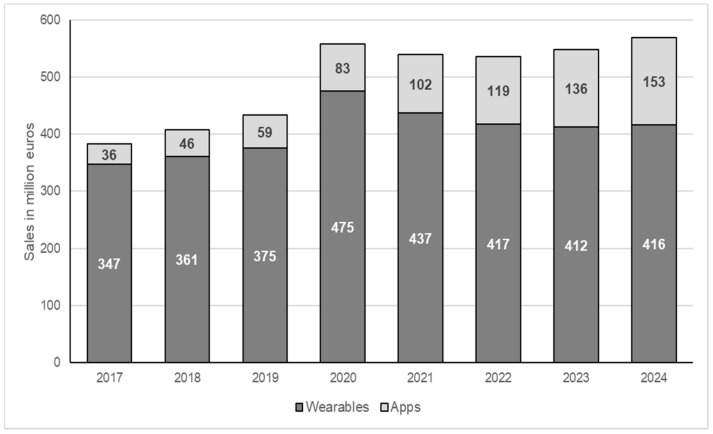
Sales of wearables and fitness apps in Germany from 2017 to 2024. Own representation based on Statista Digital Market Outlook [8].

The Statista Digital Market Outlook for Germany shows that the pandemic has strengthened the upward trend in terms of sales in the fitness tracker and fitness app sector (Figure 1). Overall, in Germany alone, this market could grow to €568 million by the end of 2024 [8].

During the pandemic, the market expanded due to new digital sports solutions that can strongly affect the exercising experience at home. In the following, we describe three best practices for digital health: the Peloton bike, the Vaha fitness mirror, and the fitness game *Ring Fit Adventure*. These have been chosen because of their high number of users and their popularity; for example, the Peloton ecosystem is being used by 2.3 million subscribers worldwide in 2021 [9].

### 2.2. Peloton Bike

The Peloton Bike is a fitness bike designed for use at home. Unlike conventional spinning bikes, it is equipped with a rotatable 24-inch HD touchscreen. In addition, the bike has Wi-Fi and Bluetooth connectivity [10]. However, the unique feature of the Peloton Bike is not its technology but its digital ecosystem, which potentially allows connecting to thousands of courses and interacting with trainers and fellow competitors. In addition to spinning classes, numerous other fitness courses, such as yoga and strength workouts, are offered [11]. 

Although there are virtual training courses, the experience is not conventional VR or AR; instead, one could say that the ecosystem creates an MR experience, as the user merges with the community of other cyclists, where the ecosystem of courses, coaches, and fellow competitors represents the “virtual” world. This combination effectively increases the motivation to achieve top performance. Accordingly, in 2020, Peloton reported sales of $1.8 billion, doubling the sales within one year, which is primarily attributable to the pandemic [12].

### 2.3. Vaha Fitness Mirror

**Figure 2:**
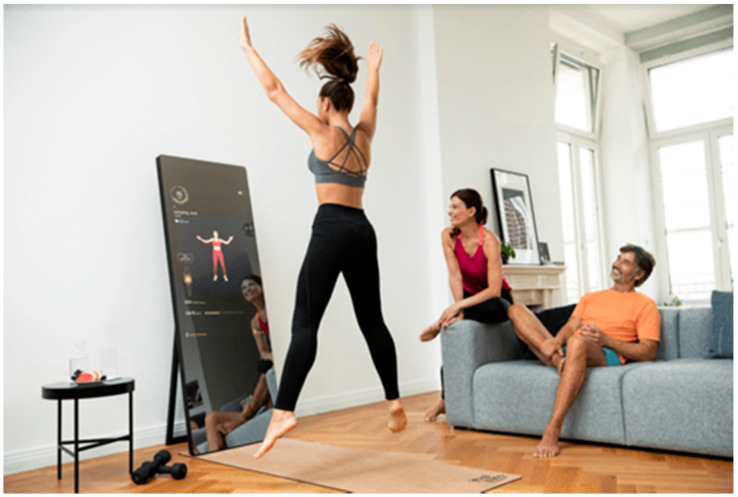
Vaha Fitness Mirror (Source: Vaha).

The Vaha fitness mirror is a workout mirror for use at home. In addition to different workouts, the mirror offers the possibility to train live with a coach via an integrated video (Figure 2). This integration creates an experience that is closest to conventional AR settings, as the coach can motivate and correct the execution of exercises. The integration of the screen in the mirror is intended to cause users to focus exclusively on themselves and the fitness experience and blend out everything else [13].

### 2.4. Nintendo Ring Fit Adventure

**Figure 3:**
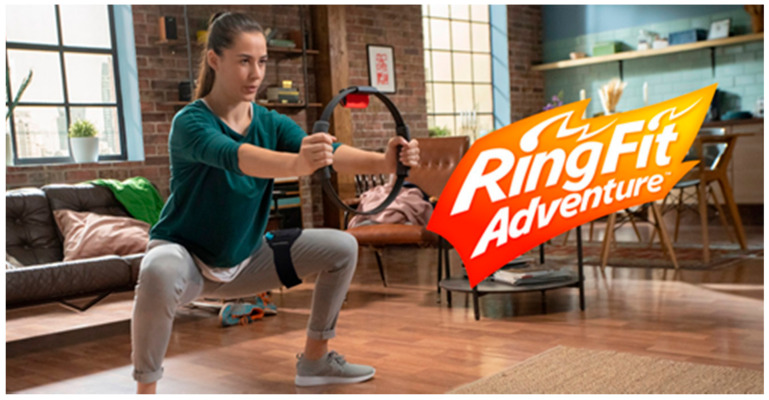
Nintendo *Ring Fit Adventure* (Source: Nintendo).

The fitness game *Ring Fit Adventure* from Nintendo is an add-on to the Nintendo Switch game console and includes a Ring-Con and a leg strap to record movements and the heart rate (Figure 3). The game’s goal is to defeat the “bodybuilder dragon” through strength and yoga exercises. *Ring Fit Adventure* combines the classic gaming experience with sports and is designed to motivate people to exercise more [14]. In this case, the device moved by the user represents the real-world anchor [4] that directly interacts with the virtual world. Unlike a regular controller, the whole body comes into play. Nintendo regularly introduces such devices; predecessors were the Balance Board from 2009 and to some extent even the Nunchuk controller for the Wii Remote shown at the 2005 Tokyo Game Show, which used an accelerometer to incorporate arm movements into games like the famous bowling game in *Wii Sports*. 

These examples show how different the starting points for digital sports can be: in one case, the base device is the bike (Peloton), while in another, the primary device is the gaming console (Nintendo Switch). The exercises in *Ring Fit Adventure* could probably be more athletic, while the Peloton courses would benefit from increased gamification. Thus, in the future, these solutions may begin to overlap and eventually even converge. The new digitized exercise experiences integrate elements from VR and AR to create an MR experience where the user is the center of attention. This focus is exemplified best by the Vaha fitness mirror, which reflects and amplifies the trend for optical self-optimization popular on apps such as *Instagram* or *TikTok*, allowing users to look at themselves while improving the body.

## 3. Related Work

In this section, we first briefly introduce basic concepts and models regarding technology acceptance, and then discuss several studies closely related to digital health as well as the effects of the pandemic in more detail.

### 3.1. Technology Acceptance

While technology can improve and facilitate everyday life, individuals are often unwilling to accept and use new systems [15]. As a consequence, technology acceptance is crucial in all fields of application and considered one of the most established streams of information system research [16]. Accordingly, several researchers have proposed competing models to assess, explain, and predict technology acceptance and adoption. Sohn and Kwon have compared the most popular technology acceptance theories to assess their suitability for AI technologies [17]. They list as the most widely used theories the technology acceptance model (TAM) [15], followed by the theory of planned behavior (TPB) [18], the unified theory of acceptance and use of technology (UTAUT) [19], and the value-based adoption model (VAM) [20].

The influential TAM investigates a system’s perceived usefulness, perceived ease of use, and users’ behavioral intention to use. The TPB adds the dimensions of behavioral control and social norms as crucial factors influencing users’ technology acceptance. The construct “social norms” summarizes the effects of social environments and influences on users’ opinion, while behavioral control refers to users’ ease of accessing a technological product. Applying the TPB as a measurement object in the context of user acceptance of innovative products found that, for their adoption, exogenous factors such as social influence play a major role [17]. Accordingly, this theory was used to explain acceptance of smart home services [21] and health cloud systems [22]. It also is a good basis for investigating digital sport solutions. 

### 3.2. Related Studies

Several recent studies show that the SARS-CoV-19 pandemic has had a negative impact on both physical activity [1,23] and mental health [24]. However, younger individuals seem to be more likely to maintain their activities than older individuals [23].

Consequently, numerous studies have investigated digital sports acceptance in the context of the pandemic. Mutz et al. (2020) describe the use of digital media for home-based sports activities [25]. This study shows that 23% of respondents used digital media for sports activities at least one time during the pandemic. Publicly available fitness videos from video platforms were used most frequently. A study from Wilke et al. (2021) also shows the increasing interest in digital home training in times of pandemic [24]. In this survey, 68% of the respondents indicated a high interest in digital home training. A third study in the context of the pandemic examined the use of digital platforms for physical activity [26]. These included digital platforms such as streaming services for exercise, subscriber fitness programs, online live or recorded classes, and electronic games. Overall, 40% of adults and 27% of adolescents reported using digital platforms for physical activity. There is considerable interest in using digital sports solutions at home in a pandemic with associated restrictions on social interactions [27].

Several studies have examined the impact of digital sports on the motivation to exercise. Most of these studies focus on fitness apps, wearables, or both. For example, some studies examine how different fitness apps increase the motivation to be active [28,29,30]. These studies unanimously support our hypotheses of positive correlations between the acceptance of digital sports solutions and real-world exercise practice.

One study [29] looks at the increasing weight of college students from the United States, which can have severe health consequences. The research examines how students use fitness apps to change their behavior. By using questionnaires, students were asked about app selection, behavioral goals, features, and reasons for continued use. Indeed, most respondents downloaded the fitness apps to achieve a personal goal, and they felt that the app had helped them achieve that goal. In addition, auditory and visual guidance were considered helpful, as they drew attention to personal achievements. 

Another study [31] also examined the motivational aspects of fitness apps. However, in this work, the focus was on social interactions. While gamification plays a role in most fitness apps, typically, individual, and competitive activities are dominant, while aspects of social interaction plays less of a role. To investigate this potential, the research team developed the game *Healthy Together*. In addition to the established individual and competitive modes, this game features a cooperation mode. The study shows that the users’ physical activity increased by 15% due to these collaborative activities. Furthermore, significantly more messages were sent in the cooperation mode. There was a positive correlation between the number of messages sent and physical activity. Based on these results, design options including cooperative features for fitness apps could be derived for practitioners [31].

As well as studies focusing on fitness apps, there is a considerable amount of studies about wearables. Several studies support the assumptions that digital sports solutions increase motivation and that wearables featuring a multitude of physiological sensors are an effective and increasingly established way to achieve this [32,33,34,35]. One study [36] explicitly compared the benefits of fitness apps and wearables. The focus was to examine helpful elements for overcoming obesity. The results show that goal setting, tracking, and feedback are the elements that most effectively increase motivation through fitness apps. For wearables, functions like reminders and rewards add additional value [36].

Other researchers [35] have examined the reasons for purchasing wearable fitness trackers with sensors. This is of importance because digital behavior tracking is controversial in other situations, such as while browsing the web or writing messages. Four focus groups discussed purchase intentions, fitness behaviors, and fitness tracker usage during the study. The two main reasons for choosing a fitness tracker were personal responsibility and affiliation. Regarding personal responsibility, the participants indicated that the wearable provided a motivating force and an ever-present reminder to exercise. Regarding belonging, the participants indicated that noticing the tracker being worn by persons close to them influenced them to participate by collaborating and competing. Gamification was another important topic, as rewards and badges were considered appealing and motivating. Competition among the group was a much-discussed topic. While competitions, such as step challenges, had a motivating effect for many, there were also opposing voices. For them, health and fitness were cooperative goals [35].

These results confirmed the findings of Chen & Pu (2014) discussed above: there should be competitive and collaborative social activities [31]. The importance of the social aspect is also highlighted by another study [37], which explored the relationship between fitness or health and social media experiences. Interestingly, many participants believed that they could improve their fitness routine by using social media. These results were confirmed by other studies [38,39] describing the motivating potential of social media for staying active.

More recent studies also point out the critical aspects of social media in connection with motivation for physical activity: One study [40] indicates that 17.4% of their participants stated that they developed an increased level of psychological stress resulting from the combination of social media and health or fitness.

In conclusion, the studies unanimously support the claim that fitness apps and wearables help achieve a healthier lifestyle. Therefore, the combination of both apps and wearables seems to be the most effective. Regarding social interactions, current solutions stress individual performance and challenges while undervaluing the potential of positive social interactions like collaborative challenges. This desire to “achieve something together” rather than “fight against each other” is likely more intense in times of social isolation, such as the current pandemic. Nevertheless, earlier research already indicates that positive social interactions are underrepresented in digital sports and health applications. 

## 4. Study

The study investigates digital sports and exercise activities: to what extent do they motivate persons to remain active in pandemic times? Based on personal experience and media coverage, we hypothesized positive correlations between the acceptance of digital sports solutions and real-world exercise practice (H1, H2, see Section 1). 

### 4.1. Method

We used a cross-sectional design survey study to identify the acceptance of digital health solutions with a particular focus on social interactions. For the purpose of this study, we created a Google Forms survey. The online questionnaire was distributed online in March and April 2021, times of complete lockdown in Germany. To quantify lockdown stringency, we determined the average Containment and Health Index (CHI, [41]). This score considers several criteria to calculate a score highlighting the severity of “lockdown” restrictions and other measures of general health protection. The average CHI over the survey distribution period (14 March to 15 April 2021) was 71.0% out of 100%, emphasizing the severity of the lockdown during this time in Germany. All participants understood the study’s aims and gave informed consent to use the data. 

The survey is divided into two sections, the first of which investigates the respondents’ sports activity before and during the pandemic. The questions are listed in Table 1.

The survey’s second part focuses on the influence of digital sports solutions on the respondents’ activity. To allow better comparability of the results, a 5-point-Likert scale (from fully disagree to fully agree) was used. The questions are listed in Table 2.

### 4.2. Sample

**Figure 4:**
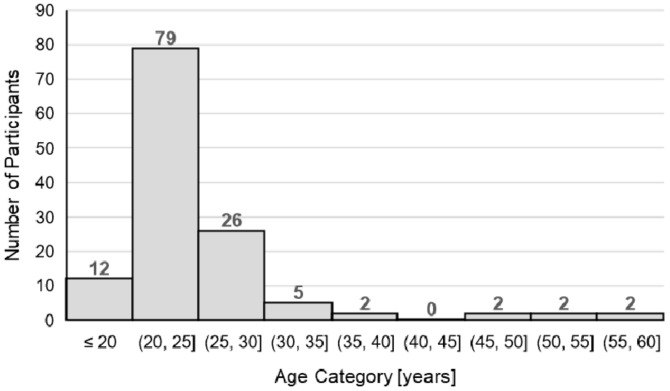
Histogram of the age distribution.

We recruited 130 participants (81 female, 49 male) via WhatsApp groups and Instagram. The sample included young academics and professionals from Germany. They were deliberately selected as potential users of digital sports solutions, most of whom were younger persons (Figure 4), with a mean age of M = 25.7 years (SD = 7.0). Only 12% of the participants did not use any digital sports solutions. The majority used multiple digital sports solutions; for example, 63% integrated social media (like YouTube) into their workouts, and 52% used fitness apps or fitness trackers. However, only 9% of the respondents used fee-based digital sport solutions.

### 4.3. Data Processing and Statistics

Descriptive statistics are reported as mean with standard deviations for each item of the survey. We used Wilcoxon rank-sum tests to analyze differences between each gender’s responses, as the data are based on ordinal scales. Differences in the time spent performing physical activity between before and during the pandemic were tested using a paired sample t-test. Due to the novelty of the pandemic situation and the relatively low sample size, we decided against alpha-level correction, since this would have reduced statistical power and increased the risk of type 2 errors. We considered the chance of overlooking a finding and thereby reducing the opportunity for further research as more harmful than potentially committing a type 1 error in this analysis. To test specific hypotheses related to the overall research question of the study, we calculated Spearman correlation coefficients between the responses to the individual questions of the survey.

## 5. Results and Discussion

In this section, we describe and discuss the study results. For some aspects, we clustered the participants according to two properties: gender and preference for team versus individual sports. For determining this preference, the participants were asked about their main sports activities (Table 1 and Table 3). The answers were classified into two groups: “mainly team sports” (*n* = 81), for example, soccer, and “mainly individual sports” (*n* = 49), for example, gym training or running. 

### 5.1. Time Spent with Physical Activity

**Figure 5:**
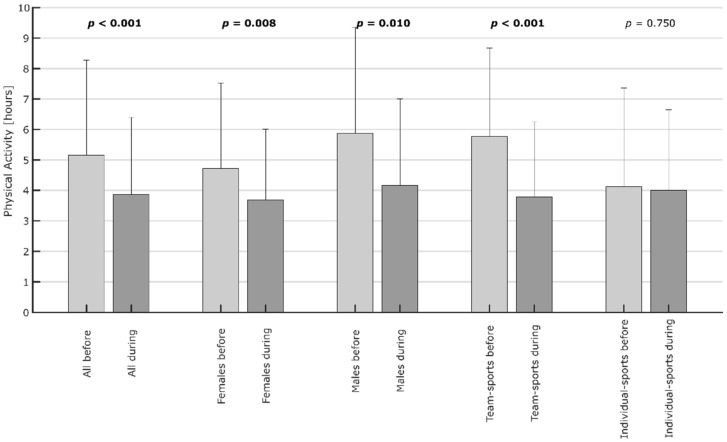
The impact of the pandemic on physical activity overall and clustered by gender and preference for individual sports versus team sports. Bold font indicates a statistically significant (*p* < 0.05) difference in physical activity between before and during the SARS-CoV-19 pandemic.

Before the pandemic, the participants (complete population) were active for M = 5.2 h (SD = 3.1 h) per week. During the pandemic, the activity dropped to M = 3.9 h (SD = 2.5 h). We observed a wide spread of values indicating the different fitness levels of the respondents. It is also noticeable that the standard deviation of physical activity before the pandemic is higher than during the pandemic (Figure 5). 

**Figure 6:**
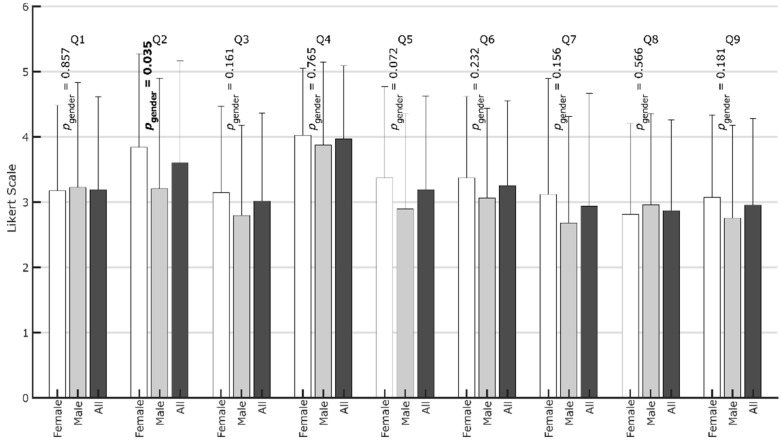
Diagram with means and standard deviations for the respective questions (Q1–Q9). The *p*-values for the tests of differences between genders are plotted vertically. Bold font indicates a statistically significant (*p* < 0.05) difference in the response between female and male participants.

The inferential data analysis shows that, on average, physical activity and sports have decreased significantly with t(129) = 5.5, *p* < 0.0000001 (Figure 6, first pair of columns). When looking at the gender dimension, differences are minor; both genders significantly reduced their sporting activity during the pandemic (Figure 6, second and third pair of columns). However, there is a striking difference between persons favoring individual sports before the pandemic, who only reduced the time spent with physical activities by 2.4% from 4.1 h (SD = 3.2 h) to 4.0 h (SD = 2.6 h), and persons preferring team sports, who reduced the time significantly by 26.9% from 5.2 h (SD = 2.9 h) to 3.8 h (SD = 2.5 h, Figure 6, fourth and fifth pair of columns). This difference is highly significant with t(81) = 4.5, *p* < 0.000001.

### 5.2. Digital Sports Acceptance

In the following, we outline the findings based on the nine survey questions. We incorporate the dimensions of gender and preference for team sports versus individual sports into our analysis. 

**Figure 7:**
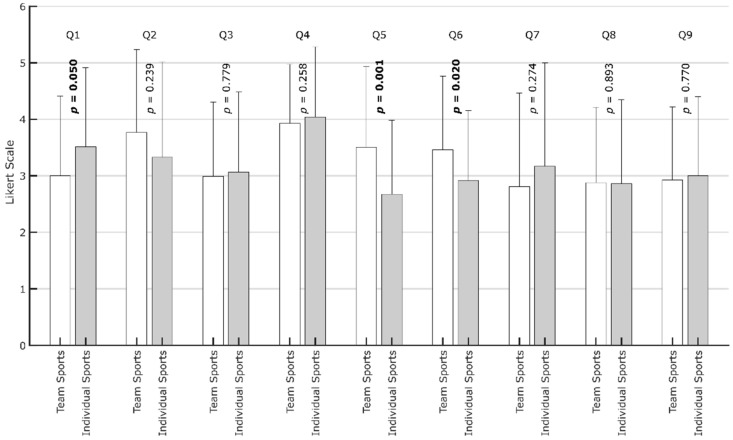
Diagram with means and standard deviations for the respective questions (Q1–Q9). The *p*-values for the tests of differences between participants who preferred team sports and those who preferred individual sports are plotted vertically. Bold font indicates a statistically significant (*p* < 0.05) difference in the response between participants with an affinity towards team sports compared to participants with an affinity towards individual sports.

Interestingly, when asked about their activity level compared to before the pandemic (Q1), most participants answered neutrally (M = 3.2, SD = 1.4, Figure 6), although the number of hours spent has been shown to be highly significantly lower. This finding shows the tendency to euphemize adverse developments during more challenging times when asked directly, stating that it is “not as bad as it looks”, though the above data show that it is. While there are few differences related to gender, the persons who preferred team sports (M = 3.5, SD = 1.5) were somewhat more aware that their behavior changed; however, the difference is on the upper end of the threshold for being significant (*p* = 0.0496, Figure 7). It is well known that team and individual athletes show different psychological traits [42,43,44,45,46]. In line with these findings, the results of this study highlight different needs between those who participate in individual and team sports for maintaining physical activity levels. This should be considered when planning digital or non-digital sports intervention programs during pandemic restrictions [27].

With M = 3.6 (SD = 1.6), the intensified use of digital sports (Q2) is apparent, although there is considerable variance. In this case, 63.8% of the participants fully agreed, while 20.8% completely disagreed. Those who fully disagreed spent on average 5.9 h (SD = 3.7 h) participating in physical activity before the pandemic and 4.0 h (SD = 3.4 h) during the pandemic, which is a drop of 32.2%. For those who fully agreed, the corresponding times only dropped from 5.2 h (SD = 3.1) to 4.1 h (SD = 2.4), equaling 21.2%. Thus, an affinity towards digital sports seems to help compensate for restricted activities. This is the only question where male and female respondents differed significantly (Figure 7): female participants were especially willing to use digital sports solutions. This result may be due to a more fitness-oriented female lifestyle, while for many men, sports still need an element of competition, which is less intense in digital sports solutions. 

When participants were asked if digital sports motivated them to stay active in the pandemic (Q3), there was some skepticism, with M = 3.0 (SD = 1.4). While 17.7% fully disagreed and 18.5% fully agreed, most were neutral. Despite these reservations regarding digital sports, the participants proved their determination to persist and not lose fitness (Q4) (M = 4.0, SD = 1.1), which were both the highest affirmation and the lowest variation. In this case, only 3.6% fully disagreed, while 38.8% fully agreed. 

When asked if online fitness challenges and live online sports in groups increased their motivation to do sports (Q5) or increased belonging (Q6), the answers are similar, with M = 3.2 (SD = 1.4; Q5) and M = 3.3 (SD = 1.3; Q6), respectively. In particular, Q6 shows that 11.5% of users fully disagreed that there is potential for social interactions, while 20.8% fully agreed. When comparing the participants preferring team sports with those preferring individual sports, the first group showed significantly higher motivation and sense of belonging due to online fitness challenges and live online sports in groups (Q5, Q6, Table 3, Figure 7). This finding further highlights the importance of considering the type of sport (individual vs. team) when thinking about countermeasures against restrictions in accessing traditional sports facilities.

When asked about the positive effects of fitness trackers (Q7), the opinion was largely neutral, with M = 2.9 (SD = 1.7). When asked about the positive effects of fitness trackers (Q7), the opinion was again largely neutral, with M = 2.9 (SD = 1.7). There were also many neutral answers regarding the positive effects of digital sports on individual performance (Q8), with M = 2.9 (SD = 1.4). Furthermore, when asked if they would continue using digital sports after the pandemic (Q9), the neutral answers, with M = 3.0 (SD = 1.3), again indicated skepticism. However, while 16.9% of the respondents fully disagreed, 14.4% said that they would continue using digital sports solutions after the pandemic. 

We explored the hypotheses regarding the use of digital sports during and after the SARS-CoV-19 pandemic using correlation analyses (Table 4).

**Hypothesis** **3** **(H3).** *Persons who stay active during the pandemic also use more digital sports solutions.*

**Hypothesis** **4** **(H4).** *Persons who already use digital sports solutions aim to continue using these in the long run.*

Overall, we found positive correlation coefficients for both hypotheses. The classification as “medium” and “large” is based on Cohen [47]. While we are aware that this classification is a subject of debate, more recent recommendations for categorization would classify all r-values above 0.3 as “large” effects [48]. 

The result of H3 are of particular note. The positive correlation between the respondents’ activity during the pandemic and the increase in motivation to remain active using digital sports solutions supports the initial assumption: people who feel motivated to remain active through digital sports solutions have been more active during the pandemic. 

The large positive correlation for H4 shows that digital sports solutions should not only be considered a temporary solution during the pandemic but also have a considerable potential for continued use after pandemic times. Some digital sports solutions, such as online challenges, could continue to be integrated into team sports for training purposes to make the sport more exciting and increase the effects of gamification. Thus, users, especially males, would be more inclined to use such solutions.

## 6. Conclusions

In this study, we looked at the impact of the pandemic on physical activity and on the acceptance of solutions for digital health and sports. Based on technology acceptance, especially the theory of planned behavior (TPB), we discussed three popular best practices in digital sports solutions: the Peloton bike, the Vaha Fitness Mirror, and the Nintendo *Ring Fit Adventure* game. Although coming from different starting points, these solutions integrate gamification and elements from VR and AR to create an experience where the user is the center of attention.

The related work unanimously supports the claim that digital sports solutions like fitness apps and wearables help to achieve a healthy lifestyle. Recent work also indicates that the general acceptance of digital sports solutions has increased during the pandemic. Regarding social interactions, current solutions stress individual performance and challenges while undervaluing the potential of positive social interactions like collaborative challenges, which would be especially important in pandemic times. 

Indeed, our evaluation of sports activity before and during the pandemic clearly shows that the pandemic has had a significant negative impact on sports activity, particularly for participants who preferred team sports before the pandemic. Most participants used more digital sports solutions than before. There is a positive correlation between the physically active time during the pandemic and the increase in motivation through digital sporting activities (r = 0.33), and many persons using digital sports aimed to continue doing so after the pandemic (r = 0.65). 

Although we found that participants were strongly determined not to decrease their overall fitness level, there was still considerable skepticism regarding the potential of digital sports solutions to increase motivation to participate in sports, improve performance, or create a sense of belonging when being physically active in groups. Male participants, in particular, seem to miss the element of direct competition found in regular team sports.

These results confirm findings from existing work and offer new insights into how the pandemic influences the acceptance of digital sports solutions and social interactions. The findings can inform the design of interventions in pandemic times as well as future digital sports solutions. Amongst others, the following practical recommendations can be made:

- The study results highlight different needs between those with preferences for individual and team sports for maintaining physical activity levels. These should be considered when planning digital or non-digital sports intervention programs during pandemic restrictions.
- Future digital sports solutions should integrate more collaborative challenges to increase both the sense of belonging through collaboration and the ability to compete with others.
- This is especially true for men and for persons who prefer team sports. Male team players were most affected by the pandemic and experienced the greatest decrease in health activities, and thus they should be especially targeted by new digital sport solutions.

### Limitations and Future Work

As indicated previously, the study sample is not representative—not even for the German population. However, the users represent a typical sample of mostly young academics and professionals who have technological affinity and sufficient income to potentially buy and use the new solutions for digital sports.

We did not perform correction of alpha levels for multiple testing. Therefore, there is a likelihood that some findings were the result of alpha-error accumulation. However, in this novel pandemic situation, we decided that increasing the risk of overlooking a potentially important finding (type 2 error) due to reducing statistical power by alpha-level adjustment may produce poorer research outcomes than strictly avoiding the risk of committing a type 1 error.

It is also important to note that the questions almost entirely focus on positive outcomes like increased motivation or personal performance. Future work should also consider the dangers posed by digital sports, particularly in combination with social media and the trend for self-optimization. Aspects such as the social pressure a regular or even permanent tracking of physiological activity can impose should be explored.

## Figures and Tables

**Table 1:** Questions regarding the sample.

S1	Before the pandemic, I spent x hours for sports activities per week. (number)
S2	During the pandemic, I spent x hours for sports activities per week. (number)
S3	Before the pandemic, I did the following sports. (free text)
S4	I use the following digital sport solutions. (free text)

**Table 2:** Survey questions.

Q1	Regardless of the restrictions during the pandemic, I am just as active in sports as I was before.
Q2	Since the start of the pandemic, I use more digital sports solutions than before.
Q3	Digital sports solutions motivate me to stay active in sports even in times of pandemic.
Q4	My goal is to use the time during lock down to not lose overall fitness level.
Q5	Online fitness challenges and live online sports in groups increase the motivation to do sports.
Q6	Online fitness challenges and live online sports in groups increase the sense of belonging in times of pandemic.
Q7	I own a fitness tracker/smart watch; by using it, I give more attention to my daily exercise.
Q8	By using digital sports solutions, I was able to improve my personal performance.
Q9	I will continue to use digital sports solutions after the pandemic.

**Table 3:** Means and standard deviations for the questions of all, female, and male participants as well as participants preferring team sports versus participants preferring individual sports.

ID	Questions (Q)	All (*n* = 130) M (SD)	Female (*n* = 81) M (SD)	Male (*n* = 49) M (SD)	Team Sports (*n* = 81) M (SD)	Individual (*n* = 49) M (SD)
Q1	Regardless of the restrictions during the pandemic, I am just as active in sports as I was before.	3.2 (1.4)	3.2 (1.3)	3.2 (1.6)	3.0 (1.4)	3.5 (1.5)
Q2	Since the start of the pandemic, I use more digital sports solutions than before.	3.6 (1.6)	3.8 (1.4)	3.2 (1.7)	3.8 (1.5)	3.3 (1.7)
Q3	Digital sports solutions motivate me to stay active in sports even in times of pandemic.	3.0 (1.4)	3.1 (1.3)	2.8 (1.4)	3.0 (1.3)	3.1 (1.4)
Q4	My goal is to use the time during lockdown to not lose overall fitness level.	4.0 (1.1)	4.0 (1.0)	3.9 (1.3)	3.9 (1.0)	4.0 (1.2)
Q5	Online fitness challenges and live online sports in groups increase the motivation to do sports.	3.2 (1.4)	3.4 (1.4)	2.9 (1.5)	3.5 (1.4)	2.7 (1.3)
Q6	Online fitness challenges and live online sports in groups increase the sense of belonging in times of pandemic.	3.3 (1.3)	3.4 (1.2)	3.1 (1.4)	3.5 (1.3)	2.9 (1.2)
Q7	I own a fitness tracker/smart watch: by using it, I give more attention to my daily exercise.	2.9 (1.7)	3.1 (1.8)	2.7 (1.6)	2.8 (1.7)	3.2 (1.8)
Q8	By using digital sports solutions, I was able to improve my personal performance.	2.9 (1.4)	2.8 (1.4)	3.0 (1.4)	2.9 (1.3)	2.9 (1.5)
Q9	I will continue to use digital sports solutions after the pandemic.	3.0 (1.3)	3.1 (1.3)	2.8 (1.4)	2.9 (1.3)	3.0 (1.4)

**Table 4:** Overview of Spearman correlation analyses between the results of selected questions.

Hypothesis	Correlation between	Correlation Coefficient
**H3**	Q1, Q3	r = 0.33 medium
**H4**	Q3, Q9	r = 0.65 large

## Data Availability

Data is available from the authors.
